# Fatty acid profile and proliferation of bovine blood mononuclear cells after conjugated linoleic acid supplementation

**DOI:** 10.1186/1476-511X-11-63

**Published:** 2012-06-05

**Authors:** Lydia Renner, Julia Pappritz, Ronny Kramer, Susanne Kersten, Gerhard Jahreis, Sven Dänicke

**Affiliations:** 1 Institute of Animal Nutrition, Friedrich-Loeffler-Institute (FLI), Federal Research Institute for Animal Health, Bundesallee 50, 38116, Braunschweig, Germany; 2 Institute of Nutrition, Friedrich Schiller University Jena, Jena, Germany

**Keywords:** CLA, Dairy cow, Peripheral blood mononuclear cells, Cell proliferation, Fatty acid profile

## Abstract

**Background:**

Conjugated linoleic acids (CLA) are in focus of dairy cattle research because of its milk fat reducing effects. Little is known about the impact of CLA on immune function in dairy cows. Therefore, in the present study we investigated the effects of a long term supplementation of dairy cows with CLA on the fatty acid profile of peripheral blood mononuclear cells (PBMC) and their proliferation ex vivo.

**Results:**

The supplementation of dairy cows with either 100 g/d of a control fat preparation (CON, n = 15), 50 g/d of the control fat preparation and 50 g/d CLA supplement – containing 12.0% *cis*-9, *trans*-11 and 11.9% *trans*-10, *cis*-12 CLA of total fatty acid methyl esters – (CLA-50, n = 15) or 100 g/d of the CLA supplement (CLA-100, n = 16) did not influence the major fatty acids (C18:0, C16:0, *cis*-9 C18:1, *cis*-9, *cis*-12 C18:2, *cis*-5, cis-8, *cis*-11, *cis*-14 C20:4) in the lipid fraction of PBMC. The proportion of *trans*-10, *cis*-12 CLA of total fatty acids was increased in both CLA supplemented groups, but there was no effect on the *cis*-9, *trans*-11 isomer. Furthermore, the proportion of *trans*-9 C18:1 and *cis*-12 C24:1 was reduced in the CLA-100 group. The mitogen stimulated cell proliferation was not influenced by CLA feeding.

**Conclusion:**

CLA supplementation influenced the FA profile of some minor FA in PBMC, but these changes did not lead to differences in the mitogen induced activation of the cells.

## Background

Conjugated linoleic acids (CLA) are a group of positional isomers of linoleic acid, which are characterized by conjugated double bonds. They are intermediate products in the biohydrogenation of unsaturated fatty acids (FA) by microorganisms in the rumen [1]. Additionally, it is reported, that CLA originate from endogenous synthesis in tissues like the mammary gland of ruminants [2]. Several positive physiological effects are reported for CLA, like anticarcinogenic (e.g. reviewed by [3,4]), antiatherogenic [5] and immunomodulatory [6] properties. In general, dietary FA are able to influence the function of immune cells due to different mechanisms, which include alteration of the membrane, changes in signal transduction pathways and in lipid mediators like Prostaglandin E_2_[7]. CLA supplementation e.g. led to decreased lymphocyte activation of healthy men [8] and declined proliferative response in rat splenocytes [9]. Dietary CLA are capable to change the FA profile of human peripheral blood mononuclear cells (PBMC), but did not alter their function, like the mitogen stimulated production of PGE_2_, leukotriene B_4_ (LTB_4_), interleukin (IL)-1β, IL-2 and tumor necrosis factor α (TNF-α) [10].

Although CLA originally occur in dairy cattle, the supplementation of the cows` diet with CLA gains in importance, because it reduces the milk fat content, which is ascribed to the *trans-*10*, cis-*12 isomer [11]. The impact of a CLA supplementation on the immune system of dairy cows has been rarely investigated. There was no effect of CLA supplementation on the stimulation index (SI) of PBMC obtained from primiparous lactating cows ex vivo 42 and 105 days *post partum* (pp). But the SI of splenocytes from the same animals were decreased following CLA supplementation [12]. It is unknown, if the effects are similar in pluriparous cows and over a longer supplementation period and if the supplementation changes the FA profile of immune cells, which might have further downstream effects. Therefore, in the present investigation the effects of a long term CLA supplementation were evaluated. In the study primiparous and pluriparous cows were involved. Effects on immune cells were evaluated by cell proliferation assays using PBMC and furthermore, the FA profile of PBMC was analyzed.

## Results

Data concerning performance of the cows in the present study are reported by Pappritz et al. [13].

### Fatty acid profile of PBMC

The main FA occurring in PBMC were C18:0 (stearic acid), C16:0 (palmitic acid), *cis-*9 C18:1 (oleic acid), *cis-*9,*cis-*12 C18:2 (linoleic acid) and *cis-*5, *cis-*8, *cis-*11, *cis-*14 C20:4 (arachidonic acid) (Table 1). CLA supplementation did not change the proportions of these FA significantly, but there was a tendency of increasing C16:0 when CLA was supplemented. Furthermore the proportion of saturated, monounsaturated and polyunsaturated FA as well as the sum of n-3 and n-6 FA were not influenced by CLA supplementation. Regarding CLA, no *trans-*10*, cis-*12 was found in PBMC of control animals, but the isomer significantly increased in both supplemented groups (Table 1). The effect was not seen for the other main supplemented isomer *cis-*9*,trans-*11, where no differences were observed between the 3 groups. Other CLA were significantly more frequently found in CON and CLA-50 group than in CLA-100 group. When all CLA isomers are considered together there were no differences among the groups. Furthermore, CLA supplementation did influence the proportion of *trans-*9 C18:1 and *cis-*15 C24:1 (Table 1). Both FA were significantly reduced in CLA-100 group compared to CON.

**Table 1:** Fatty acid profile of peripheral blood mononuclear cells

***Fatty acid***	**Group**						**Probability**
	**CON**		**CLA-50**		**CLA-100**		
C16:0	15.30	± 0.58	16.29	± 0.41	17.12	± 0.57	*0.061*
C18:0	26.81	± 1.02	27.53	± 0.82	28.18	± 0.75	0.532
C18:1*c*9	13.52	± 0.51	12.94	± 0.54	12.46	± 0.46	0.333
C18:1*c*11	2.41	± 0.13	2.25	± 0.11	2.19	± 0.08	0.338
C18:1 *t*9	0.27	± 0.01^a^	0.23	± 0.01^ab^	0.22	± 0.01^b^	**0.017**
C18:2*c*9,*c*12	10.13	± 0.23	11.07	± 0.35	10.36	± 0.27	*0.072*
CLA-*c*9,*t*11	0.17	± 0.01	0.16	± 0.01	0.15	± 0.01	*0.071*
CLA-*t*10,*c*12	0.00	± 0.00^a^	0.01	± 0.00^b^	0.02	± 0.00^b^	**<0.001**
other CLA	0.07	± 0.01^a^	0.06	± 0.01^a^	0.03	± 0.01^b^	**0.006**
C20:3n-6	3.06	± 0.14	3.07	± 0.13	2.95	± 0.16	0.805
C20:4n-6	11.12	± 0.42	9.92	± 0.49	9.96	± 0.65	0.214
C20:5n-3	1.03	± 0.08	0.96	± 0.06	0.94	± 0.05	0.553
C22:4n-6	1.35	± 0.07	1.29	± 0.08	1.27	± 0.10	0.791
C22:5n-3	4.11	± 0.29	4.03	± 0.44	4.05	± 0.22	0.295
C24:1 *c*15	0.03	± 0.01^a^	0.02	± 0.01^ab^	0.01	± 0.00^b^	**0.03**
Minor FA^†^	11.94	± 0.61	11.39	± 0.42	11.27	± 0.30	0.547
SFA	46.39	± 1.48	48.09	± 1.22	49.73	± 1.28	0.214
MUFA	20.80	± 0.691	19.78	± 0.73	18.95	± 0.48	0.131
PUFA^‡^	32.81	± 1.00	32.12	± 0.90	31.32	± 1.09	0.576
all C18:1 *trans*-FA	2.53	± 0.11	2.34	± 0.13	2.28	± 0.13	0.346
all CLA	0.24	± 0.01	0.23	± 0.02	0.20	± 0.01	*0.071*
all n-3 FA	6.37	± 0.41	6.01	± 0.42	6.10	± 0.24	0.772
all n-6 FA	26.30	± 0.71	25.98	± 0.81	25.12	± 1.00	0.602
n-3/n-6	0.24	± 0.01	0.24	± 0.02	0.25	± 0.02	0.872
MC-FA (C10 > C14)	0.53	± 0.07	0.68	± 0.10	0.87	± 0.17	0.176

Fatty acid profile of peripheral blood mononuclear cells from cows that received a control fat preparation (CON, n = 15) or 50 g/d (CLA-50, n = 15) and 100 g/d (CLA-100, n = 16) of a CLA supplement. Blood samples were taken after 70 and 140 days of supplementation and samples were pooled for each cow. Results are expressed as % of total fatty acid methyl esters, means ± standard error. p < 0.1 in italics and indicate tendencies. p <0.05 in bold and indicate significant differences, ab: different letters within a row indicate significant differences, p < 0.05, Tukey test.

^†^ Minor FA contain fatty acids which concentration is less than 1 % of all fatty acids except CLA.

^‡^ includes CLA.

c = *cis*, t = *trans*, SFA = saturated fatty acids, MUFA = monounsaturated fatty acids, PUFA = polyunsaturated fatty acids, MC-FA = medium chain fatty acid.

### Ex vivo cell proliferation assay

Cell viability and mitogen stimulated proliferation of PBMC was evaluated 7, 21, 35, 49, 105 and 182 days pp by Alamar blue (AB) and MTT assay.

**Figure 1:**
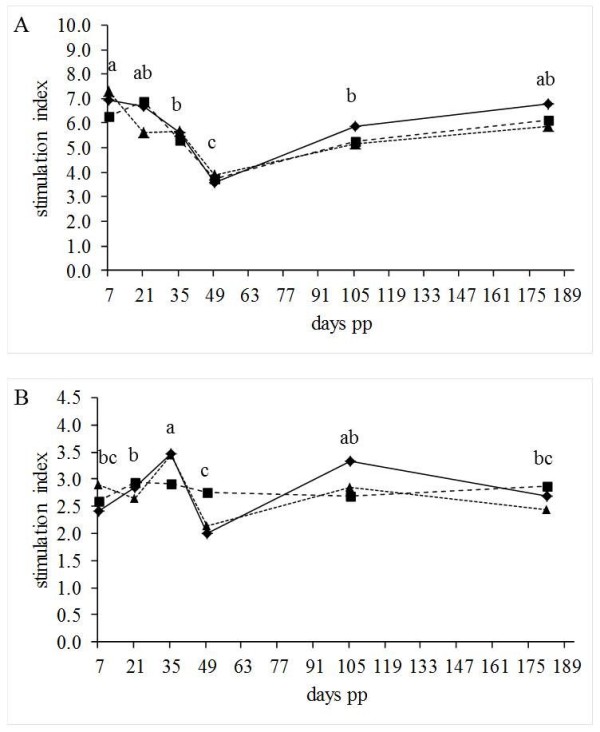
**Stimulation index of peripheral blood mononuclear cells in MTT (A) and Alamar blue assay (B).** Cows were supplemented with a control fat preparation (CON, n = 15) or 50 g/d of a CLA supplement (CLA-50, n = 15) or 100 g/d of a CLA supplement (CLA-100, n = 16), abc: indicates significant differences between sampling days, p < 0.05.

There was no effect of supplementation (p = 0.742 in MTT assay, p = 0.955 in AB assay) and lactation number (p = 0.487 in MTT assay and p = 0.972 in AB assay) on the stimulation index (SI) of PBMC. Furthermore, no interactions between day of lactation and lactation number (p = 0.948 in MTT assay and p = 0.861 in AB assay), supplementation and lactation number (p = 0.702 in MTT assay and p = 0.792 in AB assay) as well as supplementation and day of lactation (p = 0.821 in MTT assay and p = 0.128 in AB assay) were found. Only the day of lactation had a significant effect (p < 0.001 in both assays, Figure 1). At d 49 pp the SI reached the minimum in both assays. In the AB assay the SI was rising from d 7 to d 35 pp and in contrast the SI decreased from d 7 pp until d 49 pp in the MTT assay.

The SI 105 d pp was not correlated with the proportion of *cis-*9*, trans-*11 CLA, *trans-*10*, cis-*12 CLA, other CLA isomers or the sum of all CLA isomers in both assays.

## Discussion

The FA profile of immune cells is influenced by FA in the diet, thus it is possible to modify the FA profile of these cells by altering the consumption of certain FA [7]. This effect is described for n-3 polyunsaturated FA (PUFA) from fish oil or preparations of eicosapentaenoic acid or docosahexaenoic acid [14-16], but also for CLA [10] in humans. The altered FA profile of the total lipids and phospholipids, respectively, might cause changes in the function of the cells, indicated by effecting signaling pathways or the pattern of lipid mediator production [7]. Most studies were performed with humans, but also effects of certain FA on immune cell functions in dairy cows have been reported [17,18]. In these studies the effects on FA profile of bovine immune cells were not investigated [19].

In the present study the proportion of CLA in the lipid fraction of PBMC was low (less than 1% of all fatty acid methyl esters [FAME]). Due to CLA supplementation the proportion of *trans-*10*, cis-*12 CLA was increased, but the *cis-*9*, trans-*11 isomer remained unchanged. The *trans-*10*, cis-*12 isomer was not found in PBMC of the control group and it accounted for only 0.004% of total FAME in the milk fat of the same animals [13]. The *cis-*9*, trans-*11 isomer is the major CLA isomer occurring in dairy products. It is formed in the rumen by microbial fermentation [1] and by endogenous synthesis via Δ^9^-desaturase in the mammary gland [2]. These sources might have a greater impact on the proportion of *cis-*9*,trans-*11 CLA in bovine PBMC than the supplementation. There was also no effect of the diet on the percentage of *cis-*9*, trans-*11 in the milk fat of the same animals [13]. In humans, the proportion of CLA in PBMC was increased after 63 d CLA supplementation (3.9 g/d of CLA isomers). The *cis-*9*, trans-*11 isomer increased from 0.05 to 0.16% of all analyzed FA, which is in the same range as the proportion in bovine PBMC in the present study. The *trans-*10*, cis-*12 isomer increased from 0.04% to 0.19% [10], which is much higher than in the present study. In humans, the main source of CLA is the consumption of dairy products and ruminant meat [20], but the endogenous synthesis via Δ^9^-desaturase is also observed in humans, whereby *trans-*11 C18:1 serves as a precursor [21,22].

The two predominant FA in the lipid fraction of PBMC were C18:0 and C16:0 in the present study. That is in line with results from Contreras et al. [19] who investigated the FA composition of the phospholipid fraction of PBMC in dairy cows around parturition. In the present study, the major FA of the lipid fraction of PBMC were not significantly affected by CLA supplementation, but there was a trend of increased C16:0 following CLA supplementation. The increased percentage of C16:0 was also found in the FA profile of erythrocytes from new born calves whose mothers received the CLA supplement during a certain time of pregnancy [23]. Reasons for this effect are not clarified yet. CLA, particularly the *trans-*10*, cis-*12 isomer, down regulate the expression of Δ^9^-desaturase [24,25] and inhibit its activity [26] in different tissues. Therefore, a slight inhibition of Δ^9^-desaturase might be accountable for the increased proportion of C16:0. Two minor FA (<1% of total FAME), *trans-*9 C18:1 (elaidic acid) and *cis-*15 C24:1 (nervonic acid), were affected by CLA supplementation. Their percentage was decreased in CLA-100 group. Nervonic acid is an important FA in myelin sphingolipids [27] and therefore in the nervous system. Elaidic acid is, like other *trans* C:18:1 FA, mainly found in partially hydrogenated vegetable oils, but also in fat of dairy products [28]. In splenocytes obtained from rats, elaidic acid was only found in phosphatidylethanolamine and phosphatidylcholine, when elaidic acid was supplemented. In this study the elaidic acid supplementation caused increased mitogen stimulated production of interleukin-6 [29].

The mitogen stimulated proliferation was investigated to obtain information about the functionality of PBMC. The function of the cells was not influenced by the CLA supplementation, although the fatty acid composition was slightly altered in the present study. Also the milk fat depression was observed in the present study. The milk fat content was reduced dose dependently by 7% and 12% in CLA-50 and CLA-100 group, respectively, in the time period from 49 to 182 d pp [13]. Changes in the SI were observed over the lactation period. It is known that the immune system of dairy cattle is suppressed after calving [30], which was e. g. demonstrated by a decreased SI of ConA stimulated PBMC ex vivo [12]. As a reason for the immunosuppression in the pp period increased non esterified fatty acids (NEFA) concentrations, which result from increasing fat mobilization, are discussed. NEFA inhibit proliferation of bovine PBMC in vitro [31]. The immunosuppressive effect in the pp period was not observed in the MTT assay of the present study, although NEFA concentrations in plasma were much higher from 7 to 49 d pp (0.70, 0.66 and 0.69 mmol/L in CON, CLA-50 and CLA-100 group, respectively) than between d 49 and 182 pp (0.29, 0.25, 0.22 mmol/L in CON, CLA-50 and CLA-100 group, respectively) [13]. In the AB assay the SI increased from d 7 to 35 pp, but reached the minimum at d 49 pp like in the MTT assay. At that time point the cows of the CLA fed groups turned from negative to positive calculated energy balance. Until 49 d pp the cows of the CON group were not in negative calculated energy balance (14.9±5.4 MJ/d), whereas the calculated energy balance of the CLA fed cows was negative during that period (CLA-50 -12.3±5.4 MJ/d and CLA-100 -8.3±5.2 MJ/d). These differences are based on a lower dry matter (DM) intake in CLA supplemented groups during the first weeks of lactation (CON 21.1±0.7 kg/d, CLA-50 18.5±0.7 kg/d and CLA-100 17.8±0.7 kg/d). In the following period (until the end of the supplementation 182 d pp), no differences between the feeding groups occurred in DM intake (CON 21.6±0.6 kg/d, CLA-50 22.4±0.6 kg/d and CLA-100 21.2±0.6 kg/) and energy balance (CON 15.3±2.7 MJ/d, CLA-50 10.4±2.6 MJ/d and CLA-100 10.8±2.5 MJ/d) [13]. The differences between AB and MTT assay might be due to different enzyme systems involved in reduction of the respective dye. MTT is mainly reduced by mitochondrial and microsomal enzymes and AB by mitochondrial and cytosolic enzymes [32].

Hussen et al. [33] examined the leukocyte profile of PBMC of the present investigation due to CLA supplementation. Although the SI of PBMC was not influenced by CLA supplementation, there were effects on their composition. The percentage of CD4+ cells was decreased from 21 d pp onwards in the CLA-100 group compared to control and CLA-50 group and CD8+ cells were slightly increased, starting 21 d pp. The percentage of monocytes, B-cells and γδ-T cells was not altered by the diet. Furthermore, IgG1 and IgG2 levels in serum were significantly lower in the CLA-100 group throughout the supplementation period.

It is interesting to note that PBMC of calves (5 calves per group) of the CLA fed cows investigated in the present experiment showed an effect of the diet in the MTT assay (SI in CON 3.6±1.0, CLA-50 1.6±0.7, CLA-100 4.3±0.8), but not in the AB assay on mitogenic response immediately after partus and 1 day *post natum*. However, at this time, when CLA supplementation was no longer fed, the cows still did not exhibit differences in the mitogen stimulated response of PBMC, but the SI was increasing from d 0 to d 21 pp in all groups [23].

In most studies investigating the effect of CLA on immune function, the FA profiles of the investigated cells were not analyzed. Altogether, the effects of CLA supplementation to dairy cows are low and effects seen in other species, e.g. [34,35], could not be observed.

## Conclusions

Long term CLA supplementation to dairy cows did not alter the mitogen-induced proliferation of PBMC ex vivo, although the *trans-*10*,cis-*12 CLA isomer was increased in the lipid fraction of PBMC. Further investigations are necessary to evaluate if the increased proportion of *trans-*10 *cis-*12 CLA in the lipid fraction of PBMC has an impact on other immunological parameters.

## Materials and methods

### Experimental design

The experiment was carried out at the experimental station of the Friedrich-Loeffler-Institute (FLI) in Braunschweig, Germany. The study was conducted according to the European Community regulations concerning the protection of experimental animals and the guidelines of the LAVES (Lower Saxony State Office for Consumer Protection and Food Safety, Oldenburg, Germany, File number 33.14.42502-04-071/07). In the study 46 cows, 32 pluriparous and 14 primiparous, were assigned to 3 feeding groups. The control group (CON, n = 15, out of them 5 primiparous cows) received 100 g/d of a control fat preparation, the CLA-50 group (n = 15, out of them 4 primiparous cows) received 50 g/d of the control fat preparation and the CLA supplement, respectively, and the CLA-100 group (n = 16, out of them 5 primiparous cows) received 100 g/d of the CLA supplement. The supplementation period began one day pp and lasted for 182 days. During that time the cows were fed a partial mixed ration (PMR) containing 37% concentrate and 63% silage (60% maize silage, 40% grass silage based on DM content) for ad libitum consumption by a computerized feeding station (Type RIC, Insentec, B.V., Marknesse, The Netherlands). The control fat preparation (Silafat®, BASF SE, Ludwigshafen, Germany) and the CLA supplement (Lutrell® pure, BASF SE, Ludwigshafen, Germany) were given with 4 kg additional concentrate, also via a computerized concentrate feeding station. The CLA supplement contained mainly the *cis-*9*,trans-*11 and the *trans-*10*, cis-*12 isomer (12.0% and 11.9% of FAME, respectively).The daily consumption of each isomer was 4 g/d in the CLA-50 group and 8 g/d in the CLA-100 group. In the control fat preparation CLA was substituted by stearic acid, which is also the main FA in the CLA supplement. Water was offered for ad libitum consumption. More detailed information about the animal experiment, including the FA profile of the supplements, is reported elsewhere [13].

### Sample preparation

At day 7, 21, 35, 49, 70, 105, 140 and 182 pp blood (30 mL) was taken by jugular venipuncture into heparinized vacutainer tubes. PBMC were isolated from whole-blood by density gradient centrifugation using Biocoll (Biochrom AG, Berlin, Germany, L 6115). The samples were processed as described by Renner et al. [36]. Samples of day 7, 21, 35, 49, 105 and 182 pp were used to perform cell proliferation assays. The other 2 samples (70 and 140 d pp) were pooled for each cow and the FA profile of PBMC was analyzed. All samples were frozen and stored at −80°C in freezing medium containing fetal bovine serum (FBS, Biochrom AG, Berlin, Germany, S 0615) and 10% dimethyl sulfoxide (DMSO, Sigma-Aldrich, Steinheim, Germany, D 2438).

### Analysis of fatty acid profile

The PBMC were washed 3 times with saline to remove freezing medium. Cellular lipids were extracted according to the procedure described by Bligh and Dyer [37] using a methanol/chloroform mixture. The extracted lipids were then transesterificated with Boron trifluoride (BF_3_) to produce FAME, followed by a purification of the extracts using thin-layer chromatography (SIL G-25 UV_254_, Macherey-Nagel, Dueren, Germany). FAME were analyzed by gas chromatography ([GC], GC-17A Version 3, Schimadzu, Kyoto, Japan), fitted with an auto sampler and flame ionization detector. Two different procedures were necessary to identify all FAME and were conducted according to Degen et al. [38]. The general FA profile (FA, whose carbon length is 4 to 25) was analyzed using a medium polarity column (DB-225 ms, 60 m × 0.25 mm inner diameter; 0.25 μm film thickness; Agilent Technologies, Santa Clara, USA). Furthermore, the *cis* and *trans* isomers of C18:1 were separated via a high polarity column (Select^TM^ FAME, 200 m x 0.25 mm inner diameter, 0.25 μm film thickness; Agilent Technologies, Santa Clara, USA). The following reference standards were used as FAME mix to identify FA peaks: No. 463, 674, (Nu-Chek Prep, Inc., Elysian, USA), BR2, BR4, ME 93 (Larodan; Malmö, Sweden), Supelco® 37 Component FAME Mix, PUFA No. 3, conjugated linoleic acid, linoleic-, linolenic- and octadecenoic acid methyl ester mix (Supelco; Bellefonte, USA). Results are expressed as percentage of total FAME.

### Cell proliferation assays

PBMC viability and concanavalin A (ConA, Sigma–Aldrich, Steinheim, Germany, C 5275) stimulated proliferation were analyzed by MTT (3-(4,5-dimethylthiazol-2-yl)-2,5-diphenyl-tetrazolium bromide) and Alamar blue (AB) assay. The procedures were carried out as described in detail elsewhere [12].

### Calculations and statistics

Statistical analyses of the FA profile were performed by a one factorial analysis of variance (ANOVA) using the Statistica 8 for the Windows operating system, followed by a Tukey test. Probabilities below 0.05 were considered as statistically significant and p < 0.1 as a tendency.

The stimulation index (SI) was calculated by the following equation for the MTT assay:

SI = optical density (OD) of ConA stimulated PBMC/OD of non-stimulated PBMC

In the AB assay fluorescence instead of OD was used.

The PROC MIXED procedure with a compound symmetry covariance structure and supplementation, day of lactation and lactation number (primiparous vs pluriparous) as fixed factors as well as interactions of these factors was performed for statistical analyses of the SI using SAS (Software package, Version 9.1, SAS Institute, Cary, NC, USA). Because of frequent measurements during the experiment and the resulting individual cow effects, they were considered by the repeated procedure.

Correlations between the proportion of CLA isomers and the SI at 105 d pp were calculated using Statistica 8.

## Abbreviations

AB: Alamar blue; ANOVA: analysis of variance; CLA: conjugated linoleic acid; CON: control group; ConA: concanavalin A; DM: dry matter; FA: fatty acid; NEFA: non esterified fatty acids; OD: optical density; PBMC: peripheral blood mononuclear cells; PUFA: polyunsaturated fatty acids; pp: *post partum*; SI: stimulation index.

## Competing interests

The authors declare that they have no competing interests.

## Authors’ contributions

LR performed isolation of PBMC and cell proliferation assays, did statistical analysis, participated in study design and wrote the manuscript. JP carried out the animal study. RK performed fatty acid analysis of PBMC. SK helped with the statistical analysis and to draft the manuscript. GJ participated in study design and fatty acid analysis. SD participated in study design, helped with statistical analysis and to draft the manuscript. All authors read and approved the final manuscript.
